# Lipid metabolism in tumor-infiltrating regulatory T cells: perspective to precision immunotherapy

**DOI:** 10.1186/s40364-024-00588-8

**Published:** 2024-04-22

**Authors:** Yukai Shan, Tianao Xie, Yuchao Sun, Ziyi Lu, Win Topatana, Sarun Juengpanich, Tianen Chen, Yina Han, Jiasheng Cao, Jiahao Hu, Shijie Li, Xiujun Cai, Mingyu Chen

**Affiliations:** 1 Department of General Surgery, Sir Run-Run Shaw Hospital, Zhejiang University, Key Laboratory of Endoscopic Technique Research of Zhejiang Province, No.3 East Qingchun Road, 310016 Hangzhou, China; 2 National Engineering Research Center of Innovation and Application of Minimally Invasive Instruments, Sir Run-Run Shaw Hospital, Zhejiang University, 310016 Hangzhou, China; 3 School of Medicine, Zhejiang University, 310058 Hangzhou, China; 4 Department of Pathology, Sir Run-Run Shaw Hospital, Zhejiang University School of Medicine, 310016 Hangzhou, China

**Keywords:** Lipid metabolism, Tregs, Metabolic regulatory switches, Metabolic competition, Immunosuppression, Tumor therapies

## Abstract

Regulatory T cells (Tregs) are essential to the negative regulation of the immune system, as they avoid excessive inflammation and mediate tumor development. The abundance of Tregs in tumor tissues suggests that Tregs may be eliminated or functionally inhibited to stimulate antitumor immunity. However, immunotherapy targeting Tregs has been severely hampered by autoimmune diseases due to the systemic elimination of Tregs. Recently, emerging studies have shown that metabolic regulation can specifically target tumor-infiltrating immune cells, and lipid accumulation in TME is associated with immunosuppression. Nevertheless, how Tregs actively regulate metabolic reprogramming to outcompete effector T cells (Teffs), and how lipid metabolic reprogramming contributes to the immunomodulatory capacity of Tregs have not been fully discussed. This review will discuss the physiological processes by which lipid accumulation confers a metabolic advantage to tumor-infiltrating Tregs (TI-Tregs) and amplifies their immunosuppressive functions. Furthermore, we will provide a summary of the driving effects of various metabolic regulators on the metabolic reprogramming of Tregs. Finally, we propose that targeting the lipid metabolism of TI-Tregs could be efficacious either alone or in conjunction with immune checkpoint therapy.

## Background

**Fig. 1:**
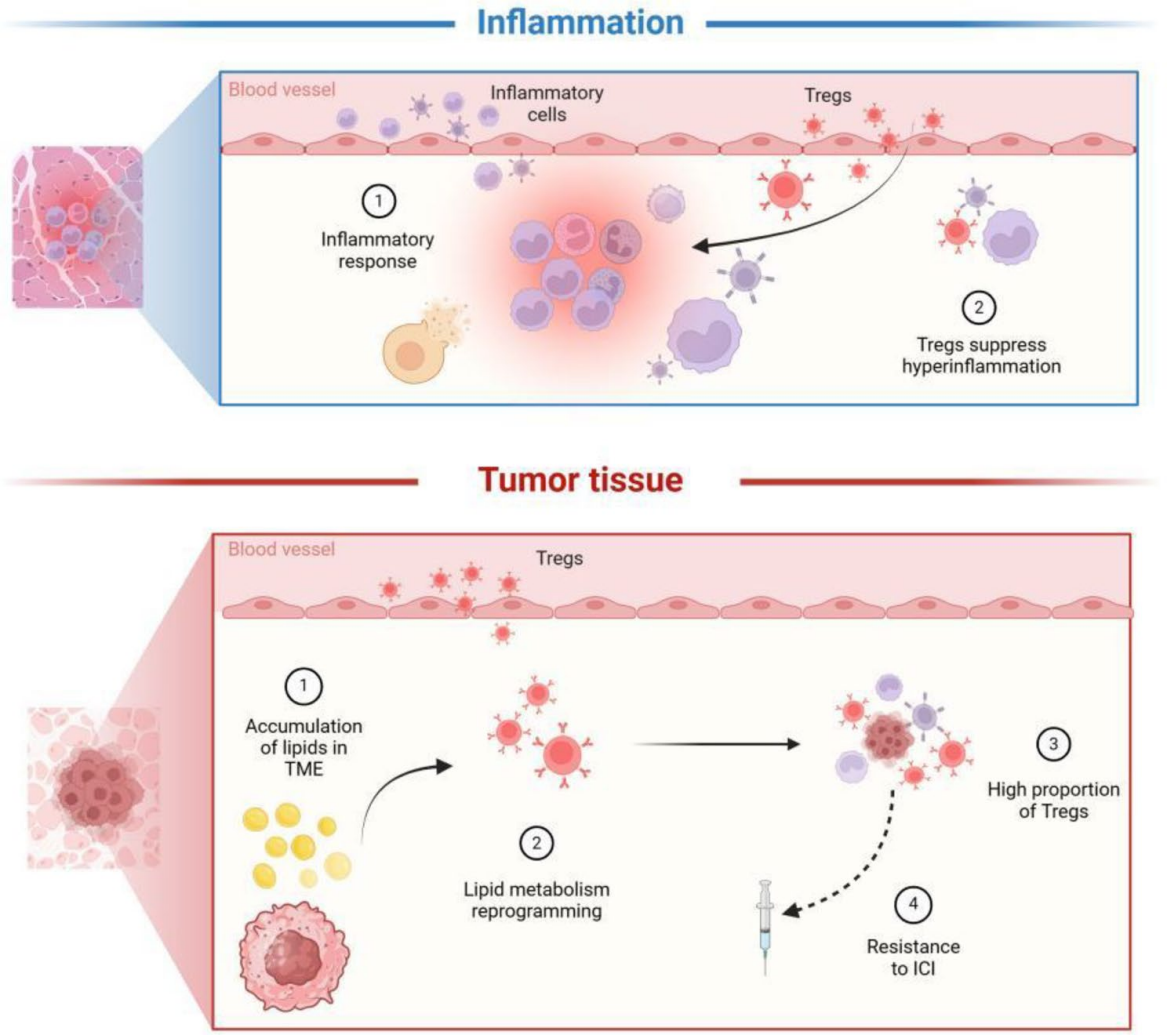
Overview of tregs. In inflamed tissues, exposure to the contents of necrotic cells induces massive infiltration of inflammatory cells to initiate the inflammatory response. During this process, Tregs that constitutively express CTLA-4, IL-2Ra, etc., are also recruited to mediate immunosuppression and prevent excessive activation of inflammation. In the tumor microenvironment(TME), the massive accumulation of lipids promotes the survival and immunosuppressive function of Tregs, leading to dysfunction of anti-tumor immune and resistance to immunotherapy. ICI: immune checkpoint inhibitor; Tregs: Regulatory T cells

Cancer is a systemic disease that compromises immune surveillance [1]. An excess of immunosuppressive activity and a deterioration of the immune surveillance system contribute to immunoincapacitation [2]. Tregs are a significant component of the immunosuppressive system that limits inflammation and maintains immune tolerance (Fig. 1). However, they are overtly stimulated within the tumor microenvironment (TME) to promote tumor development [3, 4]. Tregs elicit immune evasion from tumors via diverse mechanisms. These include inhibiting the function of antigen-presenting cells (APCs) by upregulating the expression of immune checkpoint receptors, producing inhibitory cytokines to inhibit Teffs, natural killer cells, and APCs, as well as high-affinity binding of interleukin-2 (IL-2) in TME to interfere with Teffs metabolism [5, 6].

Cancer initiation and progression require the metabolic reprogramming of cancer cells [7]. Moreover, malignant cells utilize environmental nutrients at significantly higher rates and are more flexible, thereby facilitating the formation of energy-deficient TME [8, 9]. An energy-deficient TME is characterized by extensive energy competition among immune cells, leading to energy partitioning phenomena [10]. It has been reported that lipids serve as the primary energy source for T cells in TME [11]. In contrast to Teffs, Tregs proliferate substantially within various tumor types and manifest their immunosuppressive properties [12], a mechanism that is associated with lipid metabolism and potentially pivotal to this phenomenon. Additionally, lipid accumulation is a common metabolic alteration in TME and is associated with immune dysfunction [13]. It has been discovered that Tregs upregulate forkhead box P3 (Foxp3), and surface receptors that promote metabolic adaptation, particularly lipid metabolism, to adapt to the glucose-low TME, resulting in an enhanced immunosuppressive function [14, 15].

In recent years, immunotherapies have emerged as potentially effective therapeutic alternatives, particularly for patients afflicted with advanced cancer [16]. Nevertheless, despite achieving some degree of success in preclinical investigations, the efficacy of these therapies or targets in clinical patients is limited [17]. TI-Tregs accumulation is essential for immunotherapy tolerance [6]. Notably, regulating immune metabolism significantly improves the efficacy of immunotherapy, rendering it a highly prospective therapeutic approach for cancer patients [18]. In this review, we elucidate the metabolic properties of Tregs and the lipid mediators that regulate their capacity for suppression and differentiation. Furthermore, we explore the key signaling molecules that facilitate metabolic reprogramming and how lipid signaling encourages Tregs to conform to and influence the TME, to identify potential strategies to modulate Tregs lipid metabolism and improve tumor immunotherapies.

## Lipid metabolism in regulatory T cells

The intricate interplay between the immune surveillance and tolerance systems plays a crucial role in maintaining body health against foreign pathogens or tumors [19]. Activation of the immunosuppressive system in the TME has been extensively documented in recent years, with immune metabolic reprogramming recognized as an essential factor in this phenomenon [20]. Lipid accumulation in TME is associated with immunosuppression and tumor evasion [13], which may contribute to lipid metabolic reprogramming of Tregs [14, 15].

### A brief introduction of Tregs and lipids in the TME

Tregs, characterized by specifically expressed Foxp3 transcription factor, are indispensable components of immunosuppressive function in vivo [5]. The high presence [21] and phenotype [22] of TI-Tregs have a significant impact on the prognosis of patients. Human CD4^+^ Foxp3^+^Tregs are heterogeneous in function and phenotype and can be divided into three main fractions: fraction I (Fr. I) CD45RA^+^Foxp3^low^CD25^low^ resting or naive Tregs; Fr. II CD45RA^−^FOXP3^high^CD25^high^ effector Tregs (eTregs); and Fr. III CD45RA^−^FOXP3^low^CD25^low^ cells (non-tregs) [4, 14].

Infiltration of Foxp3^hi^ Tregs (Fr. II) promotes tumor immune tolerance. Under the activation of critical developmental signals, such as CD28 costimulation signaling, TCR signaling, IL-2, or other cytokine signaling, Fr. I naive Tregs undergo differentiation into mature CD25^+^Foxp3^+^ Tregs [23, 24]. These Tregs exhibit strong suppressive activity and are commonly referred to as Fr. II effector Tregs (or mature Tregs in previous literature) [25, 26]. Fr. II effector Tregs, express HLA-DR, cytotoxic T lymphocyte antigen 4 (CTLA-4), Helios, and T-cell immunoreceptor with Ig and ITIM domains (TIGIT), have potent immunosuppressive effects [27–29]. The expression of chemokine receptors, including chemokine receptor 4 (CCR4), C–X–C chemokine receptor type 4 (CXCR4), and CXCR5, causes large infiltration of Tregs into TME [6, 14].

Recently, a consensus has emerged that the metabolic state of immune cells can significantly influence their functionality [9]. Since abnormal lipid accumulation in the TME of numerous tumors has been identified, researchers have investigated the relationship between lipid metabolism and immune competence [13, 30].

The majority of lipids in the TME contribute to immunosuppression [30]. The energy partition has been detected in the TME [10, 31], which is attributed to energy competition-dependent activation of metabolic pathways in different cells. Lipids become the primary energy source for intratumoral T cells [32, 33]. It has been reported that upregulation of lipid metabolism in Teffs effectively amplified antitumor immunity [34, 35]. However, anti-tumor immune responses are not effectively elicited in TMEs, as Tregs uptake and utilize lipids more efficiently [33, 36], resulting in increased immunosuppressive capacity. Besides, short-chain fatty acids (SCFAs) enhance the suppression function of Tregs, including upregulation of Foxp3 and CTLA-4 [37–39]–, whereas the function of long-chain fatty acids (LCFAs) remains debatable [40–42].

### Metabolic characteristics of Tregs and the underlying mechanisms

The preference of Tregs for lipid metabolism has been extensively studied in tumors [120] and adipose tissue [43]. Activation of lipid receptors peroxisome proliferator-activated receptors-γ (PPAR-γ) ameliorates adipose tissue inflammation and type 2 diabetes mellitus through transcriptional regulation of lipid metabolic programs in Tregs [44]. PPAR-γ is a crucial component in peroxisome-mediated fat acids β-oxidation (FAO) and is essential for the membrane receptor CD36 expression [43, 45]. CD36 transports LCFAs, promotes lipid β-oxidation, and fine-tunes mitochondrial fitness, which supports the stability of Tregs [46]. P38 mitogen-activated protein kinase (p38 MAPK) is a negative regulator of CD36, which blocks CD36 expression through phosphorylation of PPAR-γ [47], impairing Tregs expansion [48].

Since eTregs obtain the majority of their energy from FAO, functional mitochondria are vital for their survival [23, 49–52]. Interestingly, the mitochondrial transfer from mesenchymal stem cells to CD4^+^T cells has been shown to promote Tregs differentiation and alleviate graft-versus-host disease [23, 53]. Mitochondrial complex III [54] and mitochondrial transcription factor A [55] have been discovered to prevent DNA hypermethylation, maintaining suppressor phenotype Foxp3 expression.

Cholesterol homeostasis is essential for Tregs [33, 56, 57]. Cholesterol is an integral component of lipid metabolism as a critical component of biological processes such as biofilm components, lipoprotein components, mTORC1 activation, and immune synapses [58–60]. Increased intracellular cholesterol inhibits mTOR signaling, which favors Tregs development [61]. It has been reported that insufficient lipid availability induces upregulation of mevalonate pathway genes, which promotes intracellular cholesterol synthesis [60, 62]. The mevalonate pathway pathway-mediated protein modification is associated with aggregation of eTreg [63] and is essential for Tregs function, including the expression of PD-1 gene [33]. The hydroxy-3-methyl glutaryl coenzyme A reductase (HMGCR), a key enzyme in the mevalonate pathway, has also been shown to promote PD-1 expression [64].

## The link between lipid metabolism and immunosuppressive function of Tregs

Tregs exhibit highly heterogeneous metabolic characteristics compared with other T cells. Generally, proinflammatory cells, such as Teffs, Th17, and M1 macrophages, generate energy rapidly via glycolysis to satisfy the increased energy requirements that arise from expansion and proinflammatory activities [15]. In contrast, Tregs and memory CD8^+^ T cells mainly depend on more energy-efficient mechanisms, namely oxidative phosphorylation (OXPHOS) and FAO [15].

### Main metabolic discrepancies between Tregs and Teffs during proliferation

Glycolysis provides the energy necessary for the immense proliferation of T cells. However, specific knockdown of the glucose transporter 1 (Glut1) in T cells in vivo greatly reduces Teffs number, but conversely increases Tregs number and its suppressive capacity [18, 65, 66]. This suggests that Tregs are less dependent on glycolysis than proinflammatory T cells in vivo.

Proinflammatory cells require glycolysis at a significantly higher rate than eTregs. The PI3K-AKT-mTORC1 pathway is the major intracellular activation signal of glycolysis. In vivo administration of the mTOR inhibitor rapamycin or the glycolysis inhibitor 2-DG inhibits glycolysis and the production of IL-17, a characteristic Th17 cells cytokine, but induces the expression of Foxp3 67, CTLA-4, and TGF-β [68], increasing the proportion of Tregs. Moreover, the activation of AKT interferes with the nuclear localization of FOXO1 and down-regulates transcription of Foxp3, resulting in impaired Tregs differentiation [59]. These suggest that glycolysis activated by the PI3K-AKT-mTOR pathway in vivo is conducive to the proliferation of proinflammatory T cells [69].

Lipid metabolism is essential for Tregs proliferation. The mevalonate pathway is critical for mTOR signaling-dependent expression of suppressive molecules in Tregs [18]. Specifically, Farnesyl transferase beta subunit (Fntb) regulates mTORC1 activity and ICOS expression to maintain eTregs [63]. Protein geranylgeranyltransferase type I subunit beta (Pggt1b) acts as a regulator for TCR-dependent mTOR expression and Rac-mediated signaling to establish eTregs differentiation [63]. Furthermore, Tregs can proliferate in the absence of glucose, whereas Teffs do not. Michalek et al. demonstrated that lipid supply (but not glucose or pyruvate) reinstates Tregs generation, but not Teffs, following acute inhibition of glucose uptake [70]. Besides, it has been reported that nuclear transcription factor PPAR-γ is important to Tregs proliferation for antagonizing glycolytic WNT/β-catenin pathway and promoting lipid oxidation [44]. Administration of PPAR-γ agonist pioglitazone up-regulates the number of Tregs, thus alleviating adipose tissue inflammation in high-fat diet mice models [71]. In conclusion, glycolysis favors the proliferation of pro-inflammatory T cells, while lipid metabolism confers a proliferative advantage to Tregs.

### Lipid signaling amplifies the function of Tregs

Functionally specialized Tregs require FAO and OXPHOS. In contrast to proinflammatory T cells, which rely on glycolytic activation, central to Tregs activation is lipid metabolic reprogramming that supports their survival and function [9, 32]. Enhanced lipid oxidation and OXPHOS after TGF-β induction are critical for Tregs maturation and Foxp3 expression underlies this metabolic preference [32]. When exposed to TGF-β, PI3K-mTOR signaling is inhibited and glucose transporter is downregulated, with metabolic reprogramming to promote OXPHOS and subsequently amplifying suppressive function in eTregs [72]. In contrast, thymus-derived Tregs, express the same level of Foxp3, but are mainly dependent on glycolysis and glutamine metabolism, exhibiting weaker suppressive function [72].

**Fig. 2:**
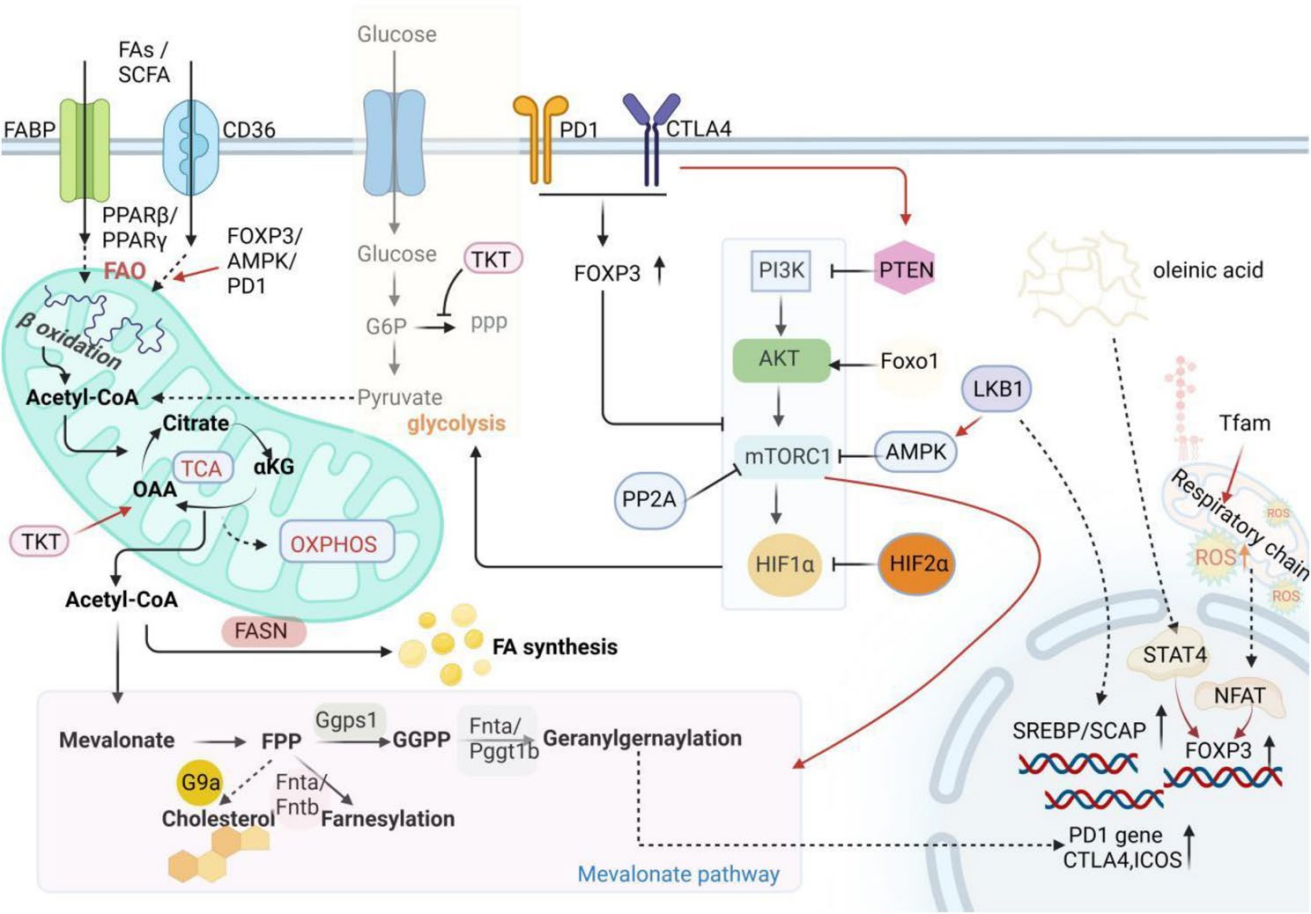
Lipid metabolism enhances treg-mediated immunosuppression. Lipid metabolism is conducive to the expression of PD-1, CTLA-4, ICOS, and FOXP3 in Tregs, enhancing immunosuppressive ability. Free fatty acids uptake enhances FAO through activating the PPAR pathway. Inhibition of PI3K-AKT-mTORC1 pathway-mediated glycolysis is conducive to the enhancement of FAO. PTEN, AMPK, PP2A, HIF2α, and FOXP3 are inhibitory molecules of the PI3K-AKT-mTORC1 pathway, which attenuate glycolysis. Oleic acids, increased reactive oxygen species levels, and PD1/CTLA-4 signal transduction are beneficial to the expression of FOXP3, inhibit glycolysis, and promote FAO. FAS is essential for the expression of immunosuppressive molecules. Here, a large amount of acetyl-coa produced by glycolysis can be used as a substrate for the synthesis of fatty acids and cholesterol, where mTORC1 has been reported to upregulate expression of CTLA-4, ICOS by activating the mevalonate pathway. Activation of the mevalonate pathway is also associated with enhanced PD-1 expression

Lipid metabolism and signaling contribute to the functional specialization of Tregs, which includes inhibition of the mTOR signaling pathway, activation of the mevalonate pathway, up-regulation of immune checkpoint PD-1, and transcription factor Foxp3 (Fig. 2). The mTOR signaling pathway plays an active role in promoting the development of Tregs, but it is downregulated in eTregs to ensure suppressive function [65]. Application of rapamycin also impinges on Teffs function, which is glycolysis dependent [68, 73]. It has been reported that Foxp3 antagonizes the PI3K-Akt-mTORC1 signaling pathway, promotes mitochondrial gene expression, and increases lipid oxidation and OXPHOS, thereby promoting functional specialization [65, 74]. Activation of protein phosphatase 2 A (PP2A), a serine-threonine phosphatase, is considered to be an essential step in the inhibition of mTORC1 complex activity mediated by Foxp3 [75]. Foxp3-mediated inhibition of Sgms1 leads to ceramide accumulation, which results in activation of the PP2A complex, providing phosphatase activity required to control mTORC1 and promoting lipid metabolism reprogramming [75]. In addition, increased FAO is beneficial to maintain intracellular fatty acids homeostasis and avoid lipotoxicity, maintaining Tregs stability.

Activation of the mevalonate pathway enhances the suppressive function of Tregs. Sterol regulatory element binding proteins (SREBPs), histone methyltransferase G9a, and the mTOR signaling pathway have been found to regulate the mevalonate pathway. The SREBPs and their cleavage activating protein (SCAP) have been indicated to be specially activated in TI-Tregs, promote mevalonate pathway-mediated lipid synthesis, especially cholesterol, which increases the expression of PD-1, and significantly reduce the efficacy of immune checkpoint therapy [33]. Furthermore, post-translational lipid modifications mediated by isoprenoid, an intermediate of the mevalonate pathway, determine the accumulation and function of eTregs [63]. Inhibition of histone methyltransferase G9a up-regulates expression of the SREBF gene (encoding SREBP), which promotes cholesterol synthesis and supports cell membrane development, resulting in enhanced induction of Tregs in intestinal inflammation [76]. Specifically, the cholesterol component of the membrane induces TGF-β autocrine signaling, leading to phosphorylation of downstream SMAD and Foxp3 expression, promoting differentiation and function of Tregs [76]. Liver kinase B1 (LKB1) is also a molecular signal for mevalonate pathway activation through coordinating AMP-activated protein kinase (AMPK)-mTOR signaling pathway or directly up-regulating expression of mevalonate pathways gene and inhibits the expression of the proinflammatory cytokine, including IFN-γ and IL-17a [77].

### The molecular basis of metabolic reprogramming in Tregs

Activated T cells undergo complex metabolic reprogramming to facilitate functional adaptation in different states [78]. The PI3K-AKT-mTOR pathway integrates various environmental cues and plays a crucial role in promoting metabolic reprogramming and maintaining physiological homeostasis. Naive CD4^+^T cells require PI3K-AkT-mTORC1-mediated glycolysis to differentiate into eTregs and proliferate [18, 79]. Conversely, enhanced glycolysis is detrimental to the suppressive function of eTregs, whereas the promotion of lipid oxidation and OXPHOS enhances its suppressive function [74]. The functional perturbation exists between the mevalonate pathway and PI3K-AKT-mTORC1 pathway and affects the physiological process of Tregs. The effect of the mTOR pathway on Tregs proliferation and function requires the mevalonate pathway [18, 58]. Upon TCR and IL-2 stimulation, mTORC1 pathway-dependent glucose metabolism provides a large number of metabolites into the mevalonate pathway for the synthesis of lipids and cholesterol, which in turn, up-regulate suppressive molecules CTLA4 and ICOS [18]. Cholesterol is a key part of the mevalonate pathway in inhibiting the mTOR pathway. Autocrine secretion of TGF-β mediated by increased membrane cholesterol content [76] and cholesterol accumulation in the cytoplasm [61] negatively regulates the mTOR pathway, and subsequently activates STAT5, which increases differentiation of Tregs and production of suppressive molecule IL-10. This implies an exquisite crosstalk between glucose metabolism and lipid metabolism.

Metabolic regulatory switches alter the activity of the PI3K-AKT-mTOR signaling pathway. The PI3K-AKT-mTOR pathway influencing factors, such as Glut1, FOXO1, AMPK, PTEN, HIF-1α, KLF10, CD36, PPAR-γ, MTHFD2, LKB1, Atg7/Atg5, and PP2A, coordinate Tregs metabolism with PI3K-AKT-mTOR pathway activity (Table 1), accompanied with functional changes in Tregs. The glycolysis up-regulated by glucose transporter Glut1 is key to maximal growth, survival, and proliferation of T cells stimulated in vitro [66]. Upon toll-like receptor (TLR) stimulation, activated PI3K-Akt-mTORC1 signaling pathway up-regulates Glut1 expression, which promotes the inflammatory function of Teffs by enhancing aerobic glycolysis, but impairs eTregs function [65]. The activation of transcription factor FOXO1 interferes with mTOR signaling and attenuates glucose metabolism, which is detrimental to the maintenance of CD4^+^T cell numbers [59]. Additionally, FOXO1 activation disrupts IL-2 signaling and maintains a low functional state of Myc against AKT activation, thereby promoting mitochondrial OXPHOS [80]. The reduction of glycolysis and mitochondrial metabolism reduces raw materials for cholesterol synthesis, subsequent reduction of mTORC1 further weakens glycolysis and CD4^+^ T cell development. The mTOR signal downstream HIF-1α has been shown to promote the proliferation of proinflammatory Th17 cells rather than Tregs in inflammation [81]. Meanwhile, HIF-1α attenuates Tregs development by promoting proteasome-dependent degradation of Foxp3 [82]. However, TME-related HIF-1α activation promotes Tregs migration, leading to poor tumor prognosis [79]. In addition, glucokinase-mediated cytoskeletal rearrangement is the key to the migration of glycolysis-dependent Tregs to inflamed tissues, inhibiting excessive inflammation [83]. The transcription factor KLF10 required to maintain mTORC1 activity has also been identified to enhance Tregs chemotaxis by promoting glycolytic reprogramming [84]. Therefore, the activation of glycolysis is considered to be a key metabolic process that promotes Tregs migration.

**Table 1:** Metabolic regulators of Tregs

Molecule	Metabolic transition	Mechanism	Function
α-KG	Enhances OXPHOS, promotes lipid storage	Up-regulates mitochondrial complex enzymes, promotes DNA methylation	Significantly attenuates Tregs differentiation and increases inflammatory cytokines [128]
FABPs	Maintain lipid metabolism and OXPHOS	Affects the integrity and function of mitochondria	Inhibition of FABP5 promotes Tregs suppressive function [32]
TKT	Stabilizes glycolysis, inhibits excessive fatty acid and amino acid catabolism	Maintains mitochondrial fitness	Maintains the suppressive function [132]
TFEB	Maintains mitochondrial function, promotes lipid metabolism	-	Increases Tregs number and suppressive function [98]
HIF-1α	Promotes glycolysis and lipid oxidation	Activates mTOR pathway; TME-related HIF1α activation prevents glucose from entering mitochondria and promotes FAO; Glycolysis drives Tregs migration	Under inflammatory conditions, HIF-1α is more prone to induce proinflammatory Teffs [67, 81, 82]; Enhances OXPHOS-dependent immunosuppression [79] Increases the number of TI-Tregs [79]
HIF-2α	—	As an inhibitory target of HIF1α	Promotes Tregs function [82]
TLR	Promotes glycolysis	Activates mTOR pathway, up-regulates Glut1	Inhibits Tregs function [65]
PTEN	Inhibits glycolysis	As an upstream inhibition target of PI3K	Inhibits the immune response induced by apoptotic tumor cell antigens and stabilizing Tregs [86]
FOXO1	Decreases glycolysis and oxidation rates, inhibits cholesterol synthesis	Activates AKT, inhibits IL-2 signaling dependent mTORC1 biosynthesis	Inhibits the proliferation of CD4^+^T cells [59]
AMPK	Inhibits glycolysis	As an mTORC1 upstream inhibitor	Increases Tregs numbers [81]
LKB1	Preserves mitochondrial function and OXPHOS, maintains cholesterol homeostasis	Activates AMPK, promotes the mevalonate pathway and its gene expression	Maintains Tregs number and function [77, 88, 89]
PP2A	Limits glycolysis	Inhibits mTORC1	Maintains suppressive function [75]
MTHFD2	Maintains purine metabolism	Maintains mTORC1 activity	Promotes Tregs differentiation [91]
SREBPs	Promote lipid and cholesterol synthesis	Activate FASN-mediated de novo fat synthesis, and inhibit mTOR signaling	Promote Tregs maturation and up-regulate the expression of PD-1 [33]
CD36	Promote lipid oxidation	Transfers LCFAs, activates PPAR-β pathway	Promotes the adaptation to TME and enhances its inhibitory function [46]
Foxp3	Inhibits glycolysis, enhances OXPHOS, and increases nicotinamide adenine dinucleotide oxidation	Inhibits mTOR and Myc	Promotes Tregs adaptation to TME and resists lactate-mediated inhibition of T cell function and proliferation [95]
SEC31A	—	Interacts with Sect. 13, activates mTORC1	Maintains the suppressive function [85]
SWI/SNF complex	—	Down-regulate amino acid sensor CASTOR1 expression, increases mTORC1 activity	Maintain the suppressive function [85]
ccdc101	—	As an inhibitor of mTORC1, limits the expression of glucose and amino acid transporters	Maintains the suppressive function [85]
Atg7/Atg5	Reduce glycolysis	Stabilize mTORC1 and c-Myc	Maintain autophagy, promote the expression of Foxp3 and suppressive function [90]
KLF10	Promotes glycolysis and mitochondrial respiration	Maintains the mTOR pathway	Maintains chemotaxis [76, 84]
G9a	Inhibits OXPHOS, enhances cholesterol synthesis	Inhibition of G9a promotes SREBP expression and the mevalonate pathway	Enhances immunosuppressive capacity [70]

Inhibitory molecules of the mTORC signaling pathway amplify Tregs suppressive function [85]. The lipid phosphatase PTEN, an upstream inhibitory target of phosphatidylinositol-3-OH kinase (PI3K), impedes antigen responses induced by apoptotic melanoma cells through enhancing PD-1 and CTLA-4 expression [86]. AMPK, upstream inhibitor of mTORC1, has been demonstrated to promote Tregs generation by attenuating glycolysis, effectively suppressing autoimmune diseases [68, 87]. The AMPK activator LKB1 is critical to Tregs stability by inhibiting the mTOR signaling pathway while activating the mevalonate pathway and maintaining cholesterol homeostasis [77]. In addition, specific loss of LKB1 leads to impaired mitochondrial function, and interferes with OXPHOS, thus impairing Tregs survival and function [88, 89]. Specific ablation of PP2A, a target that inhibits mTORC1, induces an inflammatory autoimmune immune response in vivo, because of uncontrolled glycolysis activation [75]. Foxp3 [65] and CD36 [46] activate lipid metabolism, and inhibit mTORC1 pathway activity, promoting the functional development of Tregs. Autophagy genes Atg7/Atg5 have been reported to inhibit Tregs apoptosis, thereby enhancing immunosuppressive function, the increased autophagy and attenuation of mTORC1 and Myc-mediated glycolysis mediate this process [90]. The deficiency of methylenetetrahydrofolate dehydrogenase 2 (MTHFD2), another molecule necessary to sustain the mTORC1 activity, alleviates inflammation by promoting Tregs differentiation [91]. Thus, the reprogramming of glucose metabolism mediated by the mTOR pathway to lipid metabolism and OXPHOS mediates the enhancement of immunosuppressive function in eTregs.

## TME induces lipid metabolism reprogramming of Tregs to prevail over Teffs

The immunosuppressive microenvironment is necessary for tumor growth, and tumor cells actively create a favorable microenvironment for their growth, including affecting the metabolism of immune cells [1, 92]. In general, Tregs are specifically activated and help tumor cells escape from immune surveillance, promote tumor development and distant metastasis [3]. Considerable attention has been devoted to elucidating how TI-Tregs metabolically compete with proinflammatory cells such as Th17, Th1, and Teffs, exist in large numbers, and establish an immunosuppressive environment [14]. Numerous studies have shown that the specific activation of lipid metabolism specifically promotes the survival, proliferation, and functional specialization of Tregs in the low-glucose and high-lactate TME [11, 46].

### A greater lipid uptake capacity in Tregs compared with Teffs

**Fig. 3:**
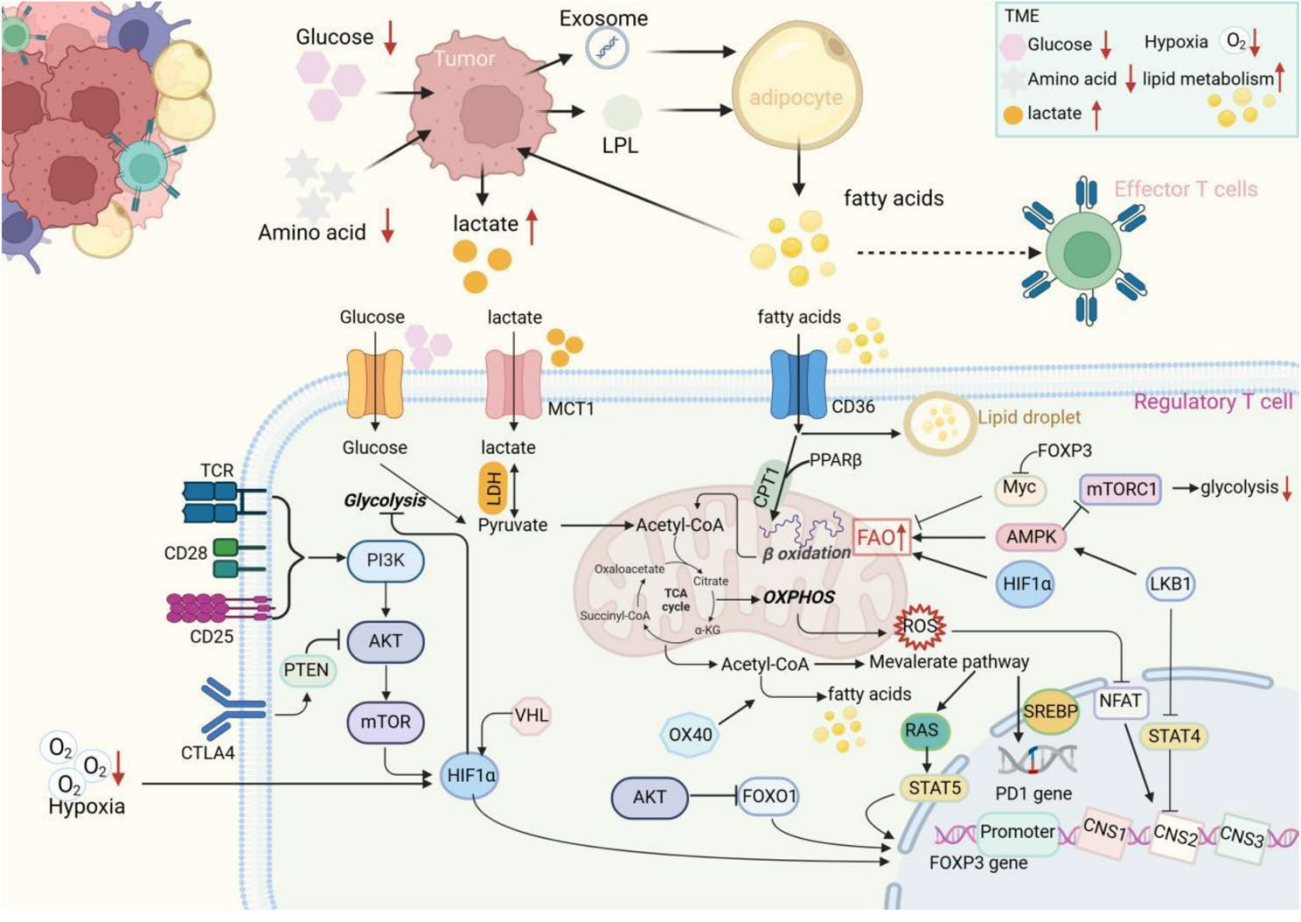
Lipid metabolism reprogramming promotes Tregs adaptation in TME. In the tumor microenvironment, most of the glucose and oxygen transported by disordered vessels are taken up by tumor cells, resulting in a hypoxia and glucose deprivation microenvironment, and the production of large amounts of lactate is detrimental to immune cell survival. Tumor-associated adipocytes are activated and promote lipid accumulation. Tregs highly express membrane receptor CD36 to uptake lipids and activate the PPAR-β pathway to enhance lipid utilization. Foxp3 expression also promotes lipid oxidation and interacts with Myc to regulate lactate dehydrogenase reaction direction, resisting the harmful effects of lactic acid accumulation. LKB1 prevents STAT4 activation-dependent CNS2 methylation of Foxp3, thereby preventing its destabilization. LKB1 also activates AMPK to inhibit mTORC1 pathway-mediated glycolysis. Under hypoxic conditions, activated HIF-1α enhances lipid metabolism and Fxop3 expression. CTLA-4 signaling activates PTEN to inhibit the mTOR pathway. Activation of the mevalonate pathway upregulates SREBP-mediated PD-1 expression, as well as RAS-STAT5-dependent Foxp3 expression

Lipid metabolism gives TI-Tregs a functional advantage. Hypoxia, glucose restriction, and high lactic acid accumulation in TME lead to widespread metabolic competition among various cells [1], which fuels metabolic reprogramming [10]. The depletion of environmental glucose and glutamine in TME drives lipid metabolic reprogramming in T cells [10, 11, 93]. It has been reported that Tregs consume free fatty acids more effectively than Teffs in cancer patients due to the greater lipid uptake capacity [12, 36], which is related to the specific expression of CD36, Foxp3, PD-1, TFEB, LKB1, CTLA-4, FOXO1, and co-stimulatory molecule OX40 (Fig. 3).

PD-1 [94] and lipid transporter CD36 [46] have been proven to mediate metabolic adaptation and intratumoral survival of TI-Tregs through activation of the PPAR-β pathway, which maintains mitochondrial lipid metabolism and increases lipid uptake. Foxp3 has been identified as another protein responsible for the upregulated lipid uptake capacity in Tregs [35]. In addition, Foxp3 expression alone is sufficient to shift the metabolic program from glycolysis to OXPHOS by inhibiting the PI3K-AKT-mTOR pathway, which enables Tregs to resist a low-glycemic, high-lactate microenvironment [35, 65], thus allowing Tregs to overwhelm conventional T cells in TME. The Myc inhibition and the FOXO1 activation are important molecular signals following the inhibition of the mTOR pathway by Foxp3^95^. Besides, Foxp3-Myc interaction influences the direction of lactate dehydrogenase (LDH) response, preventing toxicity caused by extensive accumulation of lactic acid, thereby facilitating adaptation to high-lactate TME [95]. The expression of OX40 also promotes metabolic adaptation by up-regulating lipid synthesis genes [11]. Liver kinase LKB1 contributes to Tregs survival by maintaining mitochondrial fitness and lipid metabolism, which is caused by activating the β-catenin signaling pathway to stabilize the expression of PD-1 and TNF receptor proteins, including GITR and OX40^89^. LKB1 has been reported to stabilize Foxp3 expression by preventing STAT4-mediated methylation of conserved non-coding sequence 2 (CNS2) at Foxp3 locus [96]. The mevalonate pathway upregulates Foxp3 expression through pentenylation of Ras and subsequent promotion of STAT5 phosphorylation [97]. Besides, TCR and IL2 signaling activated transcription factor TFEB specialize Tregs immunosuppressive function in tumors, because of the upregulation of lipid synthesis and metabolism genes, which favor the expression of Foxp3, CTLA-4, GITR and ICOS [98]. CTLA-4 is constitutively expressed in Tregs, which binds to CD80/CD86 costimulatory signal more efficiently than CD28 and converts the activation signal of APCs to Teffs to the activation of Tregs [99]. Upon CD80/CD86 stimulation, CTLA-4 activates PTEN, which reduces AKT phosphorylation to stabilize FOXO1, thereby activating Foxp3 expression [5]. Reducing the adverse effects of reactive oxygen species (ROS), a byproduct of OXPHOS, is an important step in the upregulation of lipid metabolism in Tregs. ROS promotes the phosphorylation of the nuclear factor of activated T cells (NFAT), preventing its nuclear translocation and blocking the binding of NFAT to the CNS2 region of Foxp3 gene, thereby inhibiting the expression of Foxp3 [100]. Glutathione [101], serine/threonine kinase 3-phosphoinositide-dependent protein kinase 1 [102], and glutathione peroxidase 4 (GPX4) [57] have been identified as key molecules in Tregs against ROS. These suggest that Tregs promote lipid metabolic reprogramming to adapt to the energy-deficient TME and promote the immune suppression function.

Furthermore, HIF-1α removes glucose from mitochondria under hypoxia TME and promotes lipid metabolism reprogramming of Tregs [79]. Thus, lipid metabolism creates the metabolic advantage for TI-Tregs to maintain immunosuppressive TME.

### Lipid accumulation promotes immunosuppression in TME

Lipid accumulation is a common metabolic disorder in TME with immune disorders [13]. Tumor cells secrete lipids or activate adipocytes contributing to lipid accumulation [12, 117]. It has been reported that RHOA mutations allow tumor cell synthesis and release of free fatty acids by amplifying PI3K-AKT-mTOR pathway-mediated glycolysis [12]. Lipid-rich TME ensures the efficient uptake and utilization of lipids that promote Tregs metabolic adaptation to facilitate their survival and function [33, 103, 104]. However, the relationship between lipid accumulation in TME and immunosuppressive microenvironment is still unclear. We highlight the critical role of Tregs in the immunosuppressive effect mediated by lipid accumulation.

**Fig. 4:**
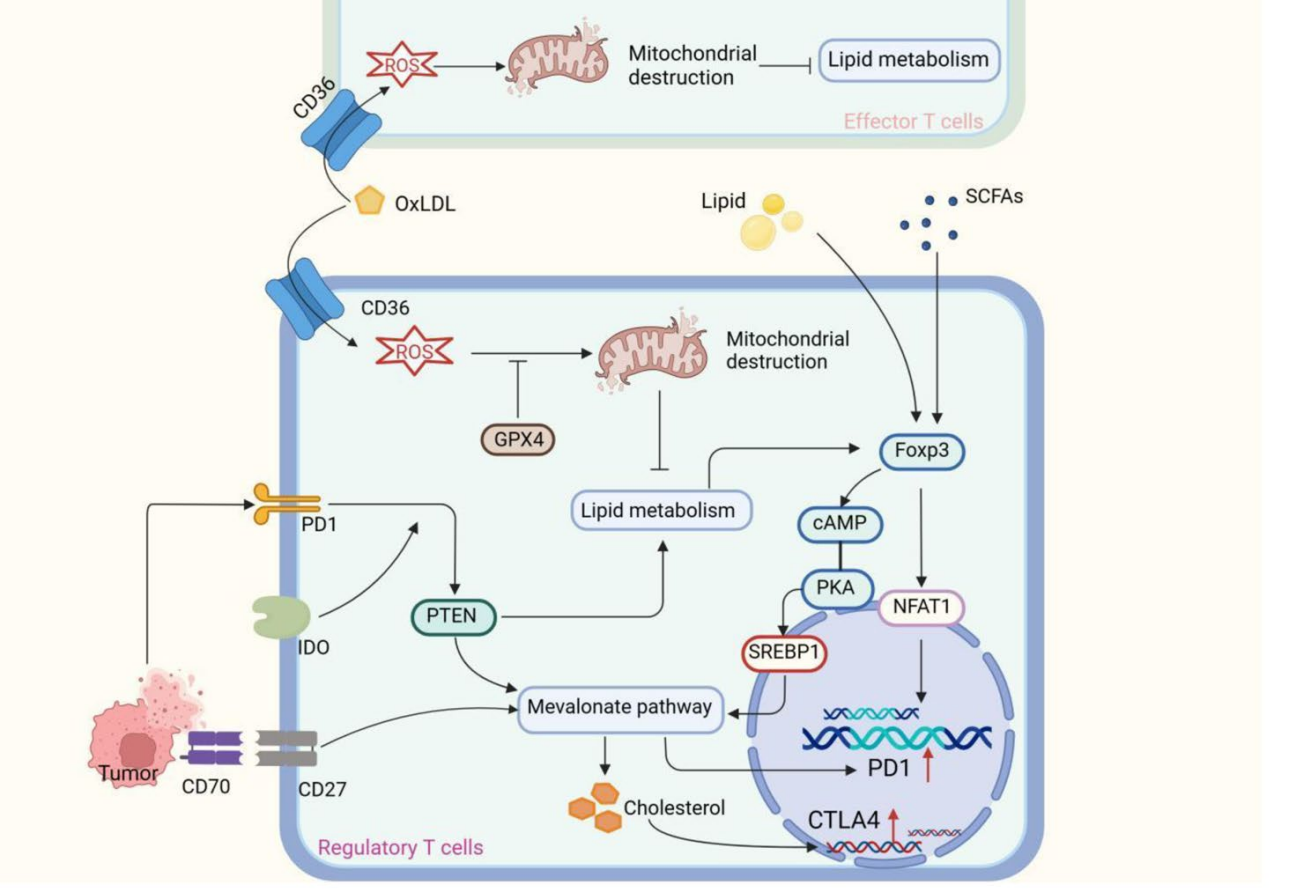
Lipid accumulation amplifies Treg suppression function. CD36 is up-regulated in both Teffs and Tregs, and the uptake of oxidized low-density lipoprotein (OxLDL) by CD36 leads to lipid peroxidation-mediated mitochondrial destruction, which interferes with metabolism and promotes cell senescence. Tregs effectively prevent lipid peroxidation by upregulating GPX4 and ensuring a high level of lipid metabolism. Apoptotic tumor cells promote mevalonate pathway-dependent cholesterol synthesis and CTLA-4 expression in Tregs by activating the PD1-PTEN signaling pathway. IDO receptor activation potentiates PD1-mediated PTEN activation. Lipids and SCFAs in TME promote the expression of Foxp3. Foxp3 promotes the expression of the PD1 gene by promoting the nuclear translocation of NFAT1. In addition, Foxp3 maintained the high level of cAMP and activated the mevalonate pathway through cAMP/PKA-SREBP1 to increase cholesterol level and CTLA-4 expression

Lipid accumulation contributes to immunosuppression in TME (Fig. 4). Lipid accumulation is detrimental to the survival of effector T cells. The intratumoral Teffs also up-regulates CD36 to promote lipid uptake but fails to adapt to TME, because of mis-ingestion of oxidized low-density lipoprotein (OxLDL), which induces lipid peroxidation, leading to Teffs dysfunction [13]. In contrast, Tregs express higher glutathione peroxidase Gpx4 than Teffs, which inhibits the formation of lipid peroxidation, thereby ensuring efficient lipid metabolism [57]. The lipid phosphatase PTEN is essential for Treg-mediated tumor immune tolerance [86, 105]. Apoptotic melanoma exposure effectively induces the expression of PTEN, which inhibits the activity of the PI3K-AKT-mTOR pathway and promotes the lipid metabolism of Tregs, thus restraining the immune response caused by apoptotic tumor cells [86]. Indoleamine 2,3-dioxygenase (IDO) is another important factor affecting PTEN activation in TI-Tregs, which is related to the activation of the PD-1 signaling pathway and the blockade of mTOR pathway, therefore PD-1→PTEN signaling maintains Tregs suppression [106].

Lipid accumulation favors PD-1 expression in TI-Tregs, thereby promoting the immunosuppressive effect by blocking costimulatory signal CD28 mediated-activation of Teffs [94], activating downstream PTEN signal [86] and lipid metabolism [94], and impeding immune checkpoint therapy [107]. Foxp3 expression, induced by lipid accumulation, enhances PD-1 expression by upregulating the expression of monocarboxylate transporter 1 (MCT1) and promoting the subsequent translocation of NFAT1 to the nucleus [107]. In addition, Foxp3 controls the direction of LDH response, promoting Tregs to adapt to a high lactate environment [95]. PD-1 maintains the Tregs suppressive capacity under chronic antigen exposure, and PD-1^cKO^ Tregs mice suffer from severe autoimmune disease due to the reduction of Tregs [94]. Interplay between Tregs and tumor-associated macrophages (TAMs) is enhanced by lipid accumulation. SREBP1-mediated lipid synthesis in TAMs, activated by Tregs down-regulating environmental IFN-γ signaling, is beneficial to its survival by maintaining mitochondrial integrity [108]. FABP5 activated in TAMs, leads to lipid accumulation and subsequent IL-10 secretion, which activates the JNK-STAT3 pathway in Tregs and promotes PD-L1 expression [109]. Tumor cells and Tregs also promote Teffs senescence by disrupting its lipid metabolism, which is associated with increased expression levels of phospholipase A2 in IVA group [110].

Cholesterol hyperaggregation is another important mechanism by which tumors establish immunosuppressive microenvironment. Compared with peripheral Tregs, the major metabolic pathway change in TI-Tregs is the upregulation of cholesterol and lipid synthesis pathways [63], which are required for their rapid proliferation and are strongly associated with suppressive phenotype, including CTLA-4 [111]. A single-cell cohort analysis of human nasopharyngeal carcinoma tissue suggests that nasopharyngeal carcinoma increases cholesterol levels of TI-Tregs by activating the mevalonate pathway and inhibiting the expression of cholesterol efflux genes through CD70-CD27 intermolecular signaling [112]. High levels of cyclic adenosine monophosphate (cAMP) may be another important cause of elevated cholesterol levels in TI-Tregs. The expression of Foxp3 in Tregs is the molecular basis for the high expression of cAMP [113], which activates its downstream protein, protein kinase A (PKA), to phosphorylate and activate SREBP1-mediated cholesterol synthesis [114, 115]. In addition, excessive intracellular cholesterol accumulation accelerates the exhaustion of Teffs. The fibroblast growth factor 21 (FGF21), secreted by tumors, activates the mTORC1-SREBP1 axis of Teffs, leading to cholesterol biosynthesis and Teffs exhaustion [116]. Besides, cholesterol accumulated in TME tends to be converted into steroids, therefore promoting immune tolerance. It has been reported that malignant cells convert cholesterol to steroids by highly expressing 11β-hydroxysteroid dehydrogenase type 1 (11β-HSD1) [117] and inducing cytochrome P450 family 11 subfamily A member 1 (CYP11A1) expression in T helper 2 cells [118], thus creating an immunosuppressive microenvironment.

Gut-derived SCFAs aggravate tumor immunosuppression. SCFAs butyrate has also been found to activate PPAR-γ and inhibit HIF-1α, promoting conversion of glycolytic metabolism to OXPHOS, thereby inducing differentiation of Tregs [119]. Gut-derived SCFAs have been found to promote regeneration and proliferation of Tregs in cancer patients, including butyrate and propionate (PA) mediated Tregs differentiation through activating Foxp3 intronic enhancer CNS1 and increasing histone H3 acetylation levels [48, 119, 120]. Lipin 2 (Lpin2) and MAPK8-interacting protein-2 (Mapk8ip2) are related genes that promote differentiation after PA treatment [121]. Increased Lpin-2 leads to downregulation of downstream JNK1-dependent proinflammatory transcription factors, and decreased phosphorylation of p38 MPAK, reducing proinflammatory T helper cells [48, 121]. Butyrate, a histone deacetylase inhibitor, enhances the stability and function of Foxp3 by promoting its acetylation, facilitating Tregs differentiation [37]. Furthermore, butyrate interacts with acetylation sites on histone H3 at the Foxp3 promoter and CNS3 promotes the expression of Foxp3 [39, 122]. Therefore, lipid accumulation specifically activates Tregs-mediated immunosuppression and is detrimental to the survival of pro-inflammatory cells.

## Targeting lipid metabolism: potential for cancer immunotherapy

The discovery of immunotherapy has revolutionized cancer therapeutics and provided a rationale for strategies to harness the immune system to fight cancer. Several types of immunotherapy, including adoptive cell transfer (ACT) and immune checkpoint inhibitors (ICIs), have demonstrated promising tumor suppressive effects, but only a small percentage of clinical cancer patients can benefit from them [17, 123]. The high frequency of TI-Tregs is a major obstacle to antitumor immunity and tumor immunotherapy [11, 95, 123]. Therefore, depleting the number of TI-Tregs or inhibiting their function would benefit tumor therapy. Although the therapeutic strategy that depletes Tregs enhances anti-tumor immunity [16], its severe side effect, namely fatal autoimmune disease, limits further application. In addition, immune checkpoint therapies targeting Tregs, such as CTLA-4 blockade, anti-PD-1 therapy, OX40, and TIGIT, have also been reported to induce myocarditis, skeletal myositis, and colitis in cancer patients, while limiting the clinical application [123]. To date, finding effective targeting methods that selectively destroy tumor-promoting Tregs remains a challenge for cancer immunotherapy [124]. Here, we show that regulating the lipid metabolism of TI-Tregs greatly attenuates intratumoral activation of Tregs and effectively impairs its suppressive effect, which will facilitate the infiltration of proinflammatory cells, and improve the efficacy of immune checkpoint therapy without significant disruption of peripheral immune homeostasis.

### Downregulation of lipid metabolism selectively inhibits Tregs

Interference with lipid metabolism inhibits the adaptation of TI-Tregs to TME. PD-1 [94]、OX40 [11]、CD36 [46]、Foxp3[95] 、CTLA-4 [125] and Gpx4 [57] have been reported to mediate the adaptation, therefore specific blocking of those processes may be beneficial to inhibit the development of tumors. Genetic or pharmacological inhibition of PD-1 in TI-Tregs impairs its function by reducing PPAR-β and SREBPs pathways dependent lipid metabolic reprogramming [94]. The activation of the mevalonate pathway is an essential cause of TCR-induced PD-1 expression on TI-Tregs [33]. Inhibiting its upstream activation molecule SREBP/SCAP or using key enzymes inhibitors of the mevalonate pathway, such as HMGCR inhibitor simvastatin, geranyl-geranyl transferase type I inhibitor GGTI and farnicyltransferase inhibitor FTI (Table 2), dramatically attenuate the immunosuppressive function through impairing lipid metabolism mediated Tregs maturation markers expression [33]. The AMPK is an intrinsic inhibitor of PD-1 expression in Tregs [64]. Activation of AMPK down-regulates the mevalonate pathway and relieves downstream p38 MAPK phosphorylation mediated inhibition of GSK3β and β-catenin axis, which interfere with PD-1 expression [64]. Therefore, the combination of AMPK agonist with anti-PD-1 antibody, anti-CTLA-4 antibody, or HMGCR inhibitor has potential clinical application. OX40, upregulated in both human and mouse TI-Tregs, is a marker of poor antitumor immunity because it promotes the FAS process in Tregs proliferation [11]. Treatment with 5-(tetradecycloxy)-2-furoic acid (TOFA), an ACC inhibitor, effectively attenuates Tregs proliferation in vitro and weakens the suppressive effect on CD8^+^T cells in vivo [11]. The glutathione peroxidase GPX4 protects TI-Tregs from lipid peroxidation and ferroptosis [57]. Specific ablation of GPX4 on TI-Tregs enhances anti-tumor immune responses, but leads to the imbalance of immunosuppression in inflammation, thereby impairing immune homeostasis [57]. Excitingly, the ablation of CD36 on Tregs effectively amplifies anti-tumor immunity without affecting Tregs functional homeostasis in the peripheral [46]. Blocking the expression of SREBP in TI-Tregs also achieves an antitumor effect without affecting the peripheral immune homeostasis [33, 94]. Therefore, targeting the molecular markers that specifically regulate the lipid metabolic reprogramming of TI-Tregs will greatly improve anti-tumor immunity without causing autoimmunity. In the future, the adverse factors of immune homeostasis caused by anti-tumor immunity should be further improved.

**Table 2:** Metabolic targeted drugs and their mechanisms of action

Target	Drug	Mechanism	Physiological effects
SREBP1	Fatostatin [108]	Inhibits lipid synthesis and mitochondrial integrity in TAMs	Attenuates Tregs-dependent TAMs
EP2\EP4	SC-560, celecoxib, AS3385282-00 (EP2i), ASP7657 (EP4i) [126]	Inhibit PGE2-EP2/EP4 signaling pathway	Reduce TI-Tregs enrichment and activation
CD70	Anti-cd70 monoclonal antibody [112]	Inhibits CD70-CD27 dependent lipid signaling network	Reduces TI-Tregs number and attenuates immunosuppression function
S1P1	Ponesimod [133, 134]	Selectively activates Akt-mTOR kinase, and promotes glycolysis	Blocks the differentiation of Tregs and inhibits the function of mature Tregs
IVA phospholipase A2	MAFP [110]	Activates MAPK, stabilizes Teffs lipids metabolism	Attenuates Treg-promoted Teffs senescence
PD-1	Anti-PD-1 antibody [33, 94]	Activates mTOR signaling pathway, attenuates FAS and cholesterol synthesis; Inhibits FAO, and reduces mitochondrial number	Attenuates TI-Tregs function
HMGCR	Adenylate [64]	Inhibits downstream GSK3β, β-catenin axis, and PD-1	Weakens suppressive function
	Simvastatin [33]	Inhibits the mevalonate pathway and activates PI3K	Detrimental to the stability of Tregs
GGTTI	GGTI [33]	Inhibits mevalonate pathway and activates PI4K	Detrimental to the stability of Tregs
Farnesyl transferase	FTI [33]	Inhibits mevalonate pathway and activates PI5K	Detrimental to the stability of Tregs
OXPHOS	Oligomycin [125]	Inhibits OXPHOS	Decreases Foxp3 expression and IL-10 production
CD36	Anti-CD36 monoclonal antibody [46]	Inhibits PPAR-β pathway, reduces lipid metabolism	Reduces TI-Tregs aggregation and function
LDH	GSK 2,837,808 A [95]	Inhibits LDL-mediated NAD consumption	Promotes the survival of Teffs and Tconv, antagonizes the effect of Tregs
CTLA-4	Ipilimumab [125]	Inhibits lipid metabolism, enhances glycolysis	Weakens suppressive capacity and the stability of TI-Tregs
HIF-1α	Acriflavine [79, 135]	Inhibits HIF-1α-mediated glycolysis that promotes Tregs migration	Disturbs TI-Tregs accumulation
PIP4K	NIH-12,848 [129]	Inhibits PI3K, mTORC1/S6 and MAPK pathways	Inhibits Foxp3 expression, impairs proliferation, and induces cell death
PTEN	VO-Ohpic [86]	Relieves the inhibition of the mTOR pathway	Promotes the conversion of Tregs to inflammatory phenotype
ACC	TOFA [11]	Reduces lipid synthesis	Inhibits proliferation and function, especially KLRG1^+^CD103^+^Tregs with high inhibitory capacity
PI3K	Wortmannin、CAL-101 [129]	Inhibits PI3K-AKT-mTOR pathway	Reduces differentiation and number
AKT	Triciribine [133]	Inhibits mTOR signaling pathway and TCR signaling	Detrimental to proliferation
mTORC1	Rapamycin [18]、Metformin [87, 128]	Inhibits mTOR signaling pathway	Reduces Tregs numbers
TCA	α-KG [112]	Promotes the methylation of Foxp3 promoter, CNS1 region	Inhibits differentiation

Breaking the signals that promote lipid metabolism improves immunosuppress microenvironment. The CD70-CD27 signaling communication between tumor and Tregs promotes tumor immunosuppressive microenvironment by enhancing lipid metabolism of Tregs [112]. Anti-CD70 mAB treatment blocks CD70-CD27 signaling, interferes with lipid metabolism, suppresses Tregs secretory profiles and expression of suppressor molecules, and significantly improves anti-tumor immunity in combination with anti-PD-1 mAB treatment [112]. VO-OHpic, an inhibitor of PTEN, relieves the inhibition of the mTOR signaling pathway, weakens lipid metabolism, and effectively promotes the activation of anti-tumor immunity after chemotherapy [86]. PGE2-EP2\EP4 pathway acts as a messenger between tumor cells and surrounding cells, helps tumor cells shape immunosuppressive microenvironment [126, 127], and also promotes ICI resistance [126]. The communication between mature DCs enriched in immunoregulatory molecules (mregDCs) and Treg has been identified as a key link in the immunosuppression driven by PGE2 signaling pathway [126]. Administration of SC-560 (COX1 inhibitor), celecoxib (COX2 inhibitor), AS3385282-00 (EP2i), and ASP7657 (EP4i) reduces PGE2 production, and inhibits PGE2-EP2\EP4 pathway activation, weakens the suppressive effect on Teffs [127], and is detrimental to recruitment and stability of TI-Tregs [126]. αKG controls lipid homeostasis and differentiation of Tregs, and supplementation of αKG significantly attenuates Tregs differentiation through promoting the methylation of Foxp3 promoter and CNS1 region [128]. It has been reported that high levels of blood SCFAs confer resistance to CTLA-4 blockade in tumor-bearing mice or clinical patients, inhibit upregulation of CD80/CD86 in APC cells, and promote Tregs activation and aggregation [38]. Thus, blocking intestinal microbiota-derived SCFAs may become a new target for anti-tumor therapy. In conclusion, targeting the lipid metabolism of TI-Tregs will be beneficial to improve the efficacy of immunotherapy and better inhibit the development and reoccurrence of tumors.

### Other therapeutic targets

Inhibition of glycolysis is advantageous for decreasing the proportion of TI-Tregs. Reprogramming of glucose metabolism improves the ability of Tregs to migrate, while cross-talk with lipid metabolism improves the suppressive phenotype of Tregs. Since HIF-1α activation promotes the migration of Tregs to the TME, specific knockout of HIF-1α in Tregs has been reported to reduce the proportion of TI-Tregs and limit tumor development [79]. The activation of the PI3K-AKT-mTORC1 signaling pathway is essential for glucose metabolism. Administration of PI3K inhibitor wortmannin, AKT inhibitor triciribine, and mTORC1 inhibitors rapamycin [18] and metformin [87], significantly reduces TI-Tregs number and function, resulting in tumor reduction. It has been shown that PIP4Ks, a lipid kinase family, is specifically elevated in TI-Tregs, and mediates the promoting effect of PI3K-AKT-mTORC1 on Tregs proliferation, application of its irreversible inhibitor NIH-12,848 greatly enhanced anti-tumor immunity [129].

Promoting the survival of anti-tumor immune cells is a promising strategy. It has been reported that Foxp3 expression is an indispensable reason for Tregs metabolic advantage in TME [95]. Interestingly, Conde et al. discovered that overexpressed Foxp3 in mature CD8^+^T cells also contributes to its survival and anti-tumor effect, increasing glucose and fatty acids uptake in energy-limited microenvironment [35]. In addition, Angelin et al. indicated that the increase of Foxp3-dependent NAD/NADH ratio is the key to the adaptation of Tregs to high lactate TME, which is related to the regulation of LDH reaction direction [95]. LDH inhibitor GSK 2,837,808 A effectively rescues the survival and proliferation ability of Teffs and Tconv by increasing the NAD/NADH ratio, which is conducive to their survival and promotes anti-tumor immunity [95]. Recently, anti-CTLA-4 treatment combined with glucose shows promising effort in oncotherapy [125]. Anti-CTLA-4 mAB ipilimumab relives CTLA-4-dependent inhibition of glycolysis, promoting glucose uptake and utilization for glycolysis, which is detrimental to Tregs stability in TME [125]. The linoleic acid treatment amplifies CD8 + T cell-mediated antitumor cytotoxicity, which is associated with enhanced lipid metabolism that reduces the activation threshold of Teffs cells [130]. The down-regulation of VLC acyl-CoA dehydrogenase (ACADVL) in Teffs is the key molecular event responsible for LCFA aggregation and lipotoxicity in TME [131]. Overexpression of ACADVL enhances mitochondrial metabolism, thus promoting Teffs intratumoral infiltration and antitumor immune responses. Metabolic blockade of TAM activation favors the tumor-killing effect of Teffs. In melanoma, Tregs maintain SREBP1-mediated lipid synthesis in M2-like TAMs by blocking proinflammatory IFN-γ production, shape immunosuppressive TME [108]. SREBP inhibitor fatostatin inhibits activation of SREBP and mevalonate pathways in M2-like TAMs, and interferes with lipid homeostasis, thus destroying mitochondrial integrity and effectively inhibiting tumor growth combined with PD-1 inhibitors [108]. Therefore, it can be met that appropriate metabolic blockade combined with immunotherapy will specifically and efficiently kill tumor cells.

## Conclusion

The crucial function of Tregs in maintaining immune homeostasis and tolerance has been brought to light by accumulating evidence in the context of the development and occurrence of autoimmune diseases and tumors. At present, there is significant research and clinical pursuit devoted to the development of antitumor strategies that specifically target Tregs, either independently or in conjunction with ICIs, chemotherapy, or cancer vaccines. Although this is the case, substantial obstacles continue to impede its true application. Severe autoimmune reactions or diseases often ensue from the ablation or suppression of Tregs at the systemic level. Consequently, in the realm of cancer immunotherapy, the search for effective methods to specifically eradicate TI-Tregs remains a formidable challenge. A vital target for regulating immune function is the connection between immune metabolism and immune function, which has been the subject of extensive research. The interplay between the glycolytic PI3K-AKT-mTOR pathway and lipid metabolism pathways, including the mevalonate pathway, facilitates metabolic reprogramming for mature Tregs to exert immunosuppression; lipid metabolic reprogramming in particular enhances this adaptation to the functional demands of Tregs at different times. Immunomodulatory molecules such as PD-1, OX40, Foxp3, and CTLA-4 play a pivotal role in promoting the adjustment of Tregs to the hyperlactate, lipid-rich, and hypoxic TME, with a particular emphasis on lipid metabolism enhancement. Anti-tumor immunotherapy will be significantly more effective if its lipid metabolism is specifically targeted at Tregs. After functional states, experimental conditions, and external stimuli, however, the impact of glucose metabolism on Tregs is extraordinarily heterogeneous. To enhance the efficacy of antitumor effects, it is critical to advance the understanding of potential mechanisms and the coordination of lipid metabolism regulation. To selectively deplete TI-Tregs in conjunction with other therapeutic approaches, additional research must focus on more effective targets.

## Data Availability

No datasets were generated or analysed during the current study.

## Abbreviations

Tregs: Regulatory T cells
eTregs: effector Tregs
TI-Tregs: tumor-infiltrating Tregs
Teffs: effector T cells
TME: tumor microenvironment
Foxp3: forkhead box P3
CTLA-4: cytotoxic T lymphocyte antigen 4
SCFAs: short-chain fatty acids
LCFAs: long-chain fatty acids
FAO: fat acids β-oxidation
OXPHOs: oxidative phosphorylation
SREBPs: sterol regulatory element binding proteins
SCAP: SREBPs cleavage activating protein
IDO: indoleamine 2, 3-dioxygenase
LDH: lactate dehydrogenase
OX40: co-stimulatory molecule
PI3K: phosphatidylinositol-3-OH kinase
AMPK: AMP-activated protein kinase
LKB1: liver kinase B1
GPX4: glutathione peroxidase 4
Glut1: glucose transporter 1
